# Achalasia: laparoscopic Heller myotomy with fundoplication versus peroral endoscopic myotomy—a systematic review and meta-analysis

**DOI:** 10.1007/s10388-024-01063-x

**Published:** 2024-05-22

**Authors:** Joana Sobral, Miguel Machado, José Pedro Barbosa, José Barbosa

**Affiliations:** 1 Faculty of Medicine, University of Porto, Alameda Prof. Hernâni Monteiro, 4200-319 Porto, Portugal; 2 Department of General Surgery, São João University Hospital Center, Porto, Portugal; 3 Department of Community Medicine, Information and Decision in Health, Faculty of Medicine, University of Porto, Porto, Portugal; 4 Department of Stomatology, São João University Hospital Center, Porto, Portugal; 5 Department of Surgery and Physiology, Faculty of Medicine, University of Porto, Porto, Portugal

**Keywords:** Achalasia, Laparoscopic Heller myotomy, POEM, Gastroesophageal reflux

## Abstract

There are various therapeutic options for achalasia. Nevertheless, peroral endoscopic myotomy (POEM) and laparoscopic Heller myotomy with fundoplication (LHM) are distinguished by their efficacy and low incidence of complications. Compare POEM and LHM regarding several outcomes in patients with achalasia. This systematic review was conducted according to the Preferred Reporting Items for Systematic Reviews and Meta-Analyses (PRISMA) guidelines. An exhaustive literature search was performed using PubMed, Web of Science, and Cochrane Library databases. Studies comparing several outcomes between POEM and LHM in patients with achalasia were included. Data on clinical success, operative time, intraoperative complications, length of stay, reintervention rates, postoperative pain, overall complications, occurrence of GERD symptoms, use of proton bomb inhibitors and esophagitis were extracted. Quality assessment of the included studies was performed using the MINORS scale. We included 20 retrospective observational studies with a combined total of 5139 participants. The results demonstrated that there was no statistically significant difference in terms of intraoperative complications, postoperative complications, reintervention rate, occurrence of GERD symptoms, GERD HRQL, use of proton pump inhibitors, and esophagitis between POEM and LHM groups. Conversely, POEM was associated with higher clinical success and shorter operative time, length of stay, and postoperative pain. This meta-analysis concludes that both POEM and LHM, are effective and safe treatments for achalasia. However, POEM demonstrates better results regarding clinical success, operative time, length of stay, postoperative pain, and a tendency towards lower recurrence.

**Supplementary Information:**

The online version contains supplementary material available at 10.1007/s10388-024-01063-x.

## Introduction

Achalasia, which is distinguished by abnormal esophageal peristalsis and ineffective relaxation of the lower esophageal sphincter (LES), is classified as a primary motor disorder of the esophagus. Although there are plenty of theories concerning its pathophysiology, the precise etiology has not been identified in its entirety [1, 2].

It is a rare disease, exhibiting an annual average incidence of 1 in 100,000 cases and a prevalence of 10 in 100,000. The incident rate escalates as age increases, with the mean age of diagnosis surpassing 50 years. Additionally, it is indifferent to ethnicity and affects both sexes [3].

All therapeutic alternatives aim to alleviate the patient's symptoms by decreasing the tone of the lower esophageal sphincter and promoting esophageal emptying. Furthermore, it is imperative that the treatments halt the disease’s progression and, as a result, avert its later complications [3].

Over the years, laparoscopic Heller myotomy with fundoplication (LHM) has been considered the gold-standard treatment due to its long-term efficacy and low associated morbidity. Since its creation in 1913, this surgical technique has come a long way from laparotomy lacking an anti-reflux mechanism to its present form, laparoscopic myotomy combined with fundoplication [4, 5].

However, in 2010, Inoue et al. published a study in which 17 adults with the diagnosis of achalasia were evaluated and subjected to POEM. There was a substantial reduction in symptomatology and LES pressure observed in all patients. By creating an endoscopic submucosal tunnel, this technique permits the extent of the dissection to be modified in accordance with the type of achalasia that has been identified. Hence, a benefit of POEM in comparison to the laparoscopic approach is the possibility for a more extensive myotomy, which could result in a significant improvement of dysphagia [6].

Consequently, over the past decade, POEM has emerged as a viable substitute for laparoscopic Heller myotomy with fundoplication due to its reduced invasiveness. Nevertheless, a concern that has been raised regarding this endoscopic method is the potential for iatrogenic gastroesophageal reflux disease to develop since POEM lacks an anti-reflux mechanism in contrast to the surgical alternative [7].

Therefore, a comparison of the outcomes of patients diagnosed with achalasia who underwent laparoscopic Heller myotomy with fundoplication or POEM is the objective of this meta-analysis. The assessed results include the efficacy of each procedure as determined by symptomatology, with the Eckardt score being the preferred measurement, the postoperative pain, the occurrence of gastroesophageal reflux following each procedure, the length of time required for procedures and hospital stays, and potential complications and relapses.

## Methods

This systematic review followed the Preferred Reporting Items for Systematic Reviews and Meta-Analyses (PRISMA) guidelines [8].

### Search strategy/information sources

Two authors independently searched the following databases for relevant literature: PubMed, Web of Science and Cochrane Library.

The search query that was entered into PubMed was: “(((Achalasia[Title/Abstract]) OR (Achalasia treatment[Title/Abstract])) AND ((Laparoscopic heller myotomy[Title/Abstract]) OR (Heller Myotomy[Title/Abstract]) OR (LHM[Title/Abstract]) OR (Surgical Myotomy[Title/Abstract])) AND ((Peroral endoscopic myotomy[Title/Abstract]) OR (POEM[Title/Abstract]) OR (Surgical Endoscopy[Title/Abstract])))”.

We used the following query in the Cochrane Library and Web of Science: “(((Achalasia) OR (Achalasia treatment)) AND ((Laparoscopic heller myotomy) OR (Heller Myotomy) OR (LHM) OR (Surgical Myotomy)) AND ((Peroral endoscopic myotomy) OR (POEM) OR (Surgical Endoscopy)))”.

Studies published from January 2010 to December 2022 were included. The selection of this time restriction was because the initial publication about POEM was published by Inoue et al. in 2010.

### Study selection and eligibility criteria

After reviewing the literature, two independent reviewers selected articles based on their titles and abstracts. Observational clinical studies comparing the results of POEM and LHM surgery in patients with achalasia were included. After deliberation among reviewers, disagreements were resolved by consensus.

Subsequently, the authors assessed the full texts and excluded articles that fit the exclusion criteria: Pediatric studies, animal studies, case reports, conference abstracts, reviews, comments, and articles not pertaining to the research question.

### Data extraction

Every original study was read and evaluated independently by two reviewers. Data extraction included study information (author, published year, study period, study design, sample size and surgical approach), patients baseline characteristics (age, gender, achalasia subtype and Eckardt score before surgery), surgical outcomes (operative time, intraoperative complications) and postoperative outcomes (length of stay, readmission rates, Eckardt score, GERD symptoms, esophagitis according to Los Angeles classification, use of proton pump inhibitors, postoperative complications according to Clavien–Dindo grade and use of analgesic medication).

### Quality assessment

The Methodological Index for Non-Randomized Studies (MINORS) checklist, comprising 12 items, was utilized by two independent reviewers to assess the quality of our included studies. A cumulative score of 24 is achievable for each study, with a score of 2 (reported and adequate), 0 (not reported), or 1 (reported but inadequate) assigned to each item specified in the checklist [9].

### Statistical analysis

We used Review Manager (Version5.4.1) to perform our data analysis. The risk ratio (RR) was used for dichotomous variables, and the mean difference (MD) was used for continuous variables. The RR and MD were considered to be statistically significant at a *p* value less than 0.05. The results of some studies were expressed as the median and range. We estimated the mean and standard deviation in accordance with the method described by Wan et al. [10]. To evaluate heterogeneity, the Cochran’s *Q* test and the *I*-squared (*I*^2^) measure were applied. We utilized a random effects model due to the heterogeneity of clinical outcomes among the included studies.

## Results

### Search results and characteristics of the included studies

**Fig. 1:**
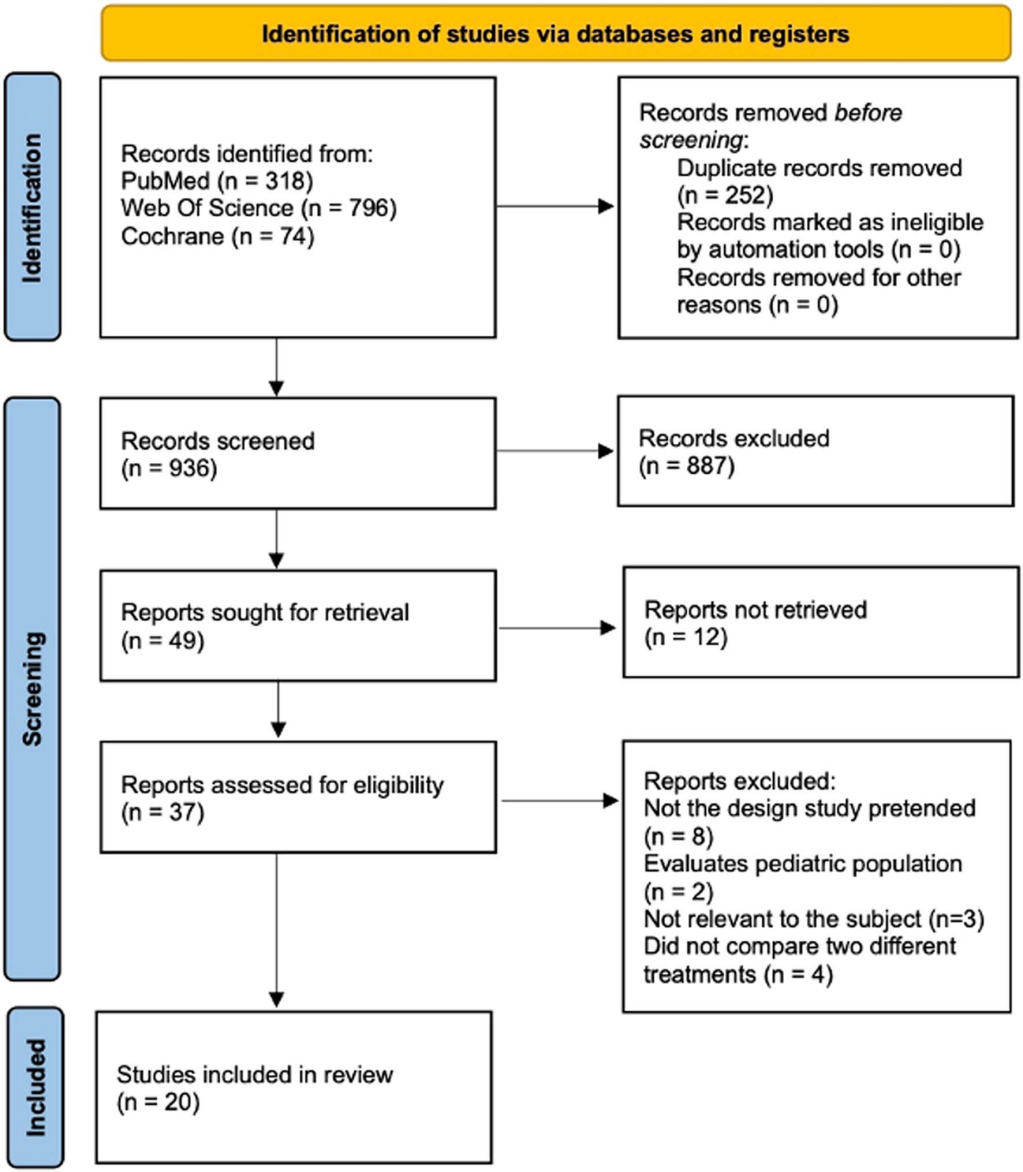
PRISMA flowchart

The initial search of PubMed, Web of Science, and the Cochrane Library platforms found 318, 796, and 74 studies, for a total of 1188 potentially relevant articles. There were 252 duplicates removed out of the total. After reading their titles and abstracts, we selected 49 full-text articles to assess for eligibility, a total of 20 studies met the eligibility criteria for the qualitative and quantitative analyses. Figure 1 presents a flowchart illustrating the reasons for excluding the remaining articles at each step of the process.

These studies include a total of 5139 participants, of which 1394 underwent POEM and 3745 underwent LHM. All studies were retrospective observational studies [11–30]. Detailed information on study characteristics and details of the patients was presented in Table 1.

**Table 1:** Summary of studies included in the meta-analysis

Authors	Year	Study design	Region	Study period	Intervention (*n*)	Comparison (*n*)	Sample size (*n*)	MINORS
Hungness et al.	2012	ROS	United States	2004–2012	*POEM* (*n* = 18)	*LHM* (*n* = 55)	73	17
Bhayani et al.	2014	ROS	United States	2007–2012	*POEM* (*n* = 37)	*LHM* (*n* = 64)	101	19
Kumbhari et al.	2014	ROS	Multinational	2000–2013	*POEM* (*n* = 49)	*LHM* (*n* = 26)	75	16
Chan et al.	2016	ROS	China	2001–2014	*POEM* (*n* = 33)	*LHM* (*n* = 23)	56	17
Docimo et al.	2016	ROS	United States	2006–2015	*POEM* (*n* = 44)	*LHM* (*n* = 122)	166	20
Schneider et al.	2016	ROS	United States	2004–2016	*POEM* (*n* = 25)	*LHM* (*n* = 25)	50	18
Ward et al.	2017	ROS	United States	2011–2016	*POEM* (*n* = 41)	*LHM* (*n* = 24)	65	18
Leeds et al.	2017	ROS	United States	2014–2017	*POEM* (*n* = 12)	*LHM* (*n* = 11)	23	18
de Pascale et al.	2017	ROS	Italy	2012–2015	*POEM* (*n* = 32)	*LHM* (*n* = 42)	74	17
Peng et al.	2017	ROS	China	2009–2012	*POEM* (*n* = 13)	*LHM* (*n* = 18)	31	20
Ramirez et al.	2017	ROS	Argentina	2010–2016	*POEM* (*n* = 35)	*LHM* (*n* = 35)	70	16
Shea et al.	2019	ROS	United States	2009–2018	*POEM* (*n* = 44)	*LHM* (*n* = 97)	141	17
Shemmeri et al.	2019	ROS	United States	2005–2017	*POEM* (*n* = 71)	*LHM* (*n* = 114)	185	16
Wirsching et al.	2019	ROS	United States	2014–2017	*POEM* (*n* = 23)	*LHM* (*n* = 28)	51	19
Ward et al.	2020	ROS	United States	2015–2019	*POEM* (*n* = 54)	*LHM* (*n* = 46)	100	19
Attar et al.	2020	ROS	United States	2010–2020	*POEM* (*n* = 126)	*LHM* (*n* = 33)	159	18
Kahaleh et al.	2020	ROS	Latin America	2015–2020	*POEM* (*n* = 69)	*LHM* (*n* = 64)	133	20
Podboy et al.	2020	ROS	United States	2010–2015	*POEM* (*n* = 55)	*LHM* (*n* = 43)	98	17
Trieu et al.	2021	ROS	United States	2013–2017	*POEM* (*n* = 580)	*LHM* (*n* = 2850)	3430	19
Shally et al.	2022	ROS	United States	2014–2021	*POEM* (*n* = 33)	*LHM* (*n* = 25)	58	17

*LHM* laparoscopic heller myotomy; *POEM* peroral endoscopic myotomy; *ROS* retrospective observational study

### Quality assessment

The median score in the MINORS scale was 18, with a range of 16–20, as indicated in Table 1. As a result, all included studies were deemed adequate for inclusion in the quantitative analysis.

### Surgical outcomes

Operative time was reported by twelve studies, involving a total of 892 patients. It was significantly shorter in the POEM group, when compared with the LHM. Mean operation time was 116.8 min in the POEM group and 150.5 min in the LHM group. (MD −33.80, *p* < 0.00001; 95% CI: −46.07, −21.53). There was high heterogeneity among studies (*P*_heterogeneity_ < 0.00001, *I*^2^ = 90%). We conducted sensitivity analysis to explore the sources of heterogeneity and identified only one study with a different result from the rest. The sole study that reveals a longer operative time in POEM explains this through the learning curve associated with the technique [16].

Eight studies reported intraoperative complications. The rate of intraoperative complications was 11.1% (40/359) in the POEM group and 8.94% (27/302) in the LHM group. This difference was not statistically significant (RR 1.21, *p* = 0.48; 95% CI 0.72–2.04) with low heterogeneity (*P*_heterogeneity_ = 0.44, *I*^2^ = 0%).

### Postoperative outcomes

In the included studies, clinical success was defined as an Eckardt score of three or lower. It was reported in eight studies. Clinical success rate was 91.2% (250/274) in the POEM group and 82.3% (246/299) in the LHM group. The POEM group had a significantly higher clinical success when compared with the LHM (RR 1.08, *p* = 0.010; 95% CI: 1.02, 1.14) with low heterogeneity (*P*_heterogeneity_ = 0.76, *I*^2^ = 0%).

Ten studies reported the postoperative Eckardt score's mean and standard deviation instead of using the clinical success criterion of being equal to or less than 3. Mean postoperative Eckardt scores was 1.41 in the POEM group and 1.49 in the LHM group. There was no statistically significant difference between the POEM group and the LHM group (MD −0.08, *p* = 0.76; 95% CI: −0.63, 0.46). There was high heterogeneity among studies (*P*_heterogeneity_ < 0.00001, *I*^2^ = 86%).

Regarding postoperative complications, the overall complication rate was 7.7% (91/1178) in the POEM group and 5.7% (195/3431) in the LHM group. The meta-analysis did not demonstrate a statistically significant difference among the 15 studies that reported this outcome (RR 0.89, *p* = 0.62; 95% CI: 0.57, 1.40) with low heterogeneity (*P*_heterogeneity_ = 0.04, *I*^2^ = 43%).

Ten studies reported postoperative complications corresponding to CD grades I and II. This rate was 5.4% (52/971) in the POEM group and 4.2% (130/3114) in the LHM group. This difference was not statistically significant (RR 0.78, *p* = 0.34; 95% CI: 0.47, 1.30) with low heterogeneity (*P*_heterogeneity_ = 0.10, *I*^2^ = 39%).

Surgical complications corresponding to CD grade III were included in thirteen studies and demonstrated no statistically significant difference between both groups. In the POEM group, it was 2.7%, and in the LHM group, it was 1.1% (RR 1.20, *p* = 0.82; 95% CI: 0.26, 5.48) with low heterogeneity (*P*_heterogeneity_ = 0.20, *I*^2^ = 37%).

Surgical complications corresponding to CD grade IV were reported in three studies and demonstrated no statistically significant difference between the two groups. In the POEM group, it was 2.8%, and in the LHM group, it was 1.2% (RR 1.18, *p* = 0.77; 95% CI: 0.40, 3.43) with low heterogeneity (*P*_heterogeneity_ = 0.18, *I*^2^ = 39%).

Regarding reintervention rate, seven studies reported this outcome, and reintervention rate was comparable between both groups. Reintervention rate was 10.7% (36/335) in the POEM group and 17.4% (58/334) in the LHM group (RR 0.62, *p* = 0.06; 95% CI: 0.37, 1.02) with low heterogeneity (*P*_heterogeneity_ = 0.20, *I*^2^ = 29%).

Through the duration of analgesic medication use, postoperative pain was evaluated in two studies. According to our research, the POEM group experienced significantly less pain following surgery. Mean duration of analgesic medication use was 1.6 days in the POEM group and 2.3 days in the LHM group (MD −0.91, *p* = 0.03; 95% CI: −1.71, −0.11) with low heterogeneity (*P*_heterogeneity_ = 0.64, *I*^2^ = 0%).

Length of stay (LOS) was reported in fourteen studies. The mean length of hospital stay was 2.1 days in the POEM group and 2.7 days in the LHM group. The POEM group had a significantly lower length of stay (MD −0.55, *p* = 0.0001; 95% CI: −0.83, −0.27). However, there was high heterogeneity among studies (*P*_heterogeneity_ < 0.00001, *I*^2^ = 83%).

Regarding gastroesophageal reflux, six studies reported the occurrence of GERD symptoms. The rate of GERD symptoms was 21% (43/204) in the POEM group and 18.1% (40/221) in the LHM group. There was no statistically significant difference. (RR 0.94, *p* = 0.75; 95% CI: 0.37, 1.02) with low heterogeneity (*P*_heterogeneity_ = 0.72, *I*^2^ = 0%).

Four studies applied the GERD HRQL questionnaire both prior to and following the procedures. After the procedures, the mean of these questionnaires was comparable between the groups (MD 1.46, *p* = 0.13; 95% CI: −0.41, 3.33) with low heterogeneity (*P*_heterogeneity_ = 0.58, I^2^ = 0%).

Five studies assessed the postoperative use of proton pump inhibitors. PPI use was 25.4% (36/184) in the LHM group and 25.5% (47/184) in the POEM group. There is no statistically significant difference between both groups (RR 0.92, *p* = 0.65; 95% CI: 0.64, 1.31) with low heterogeneity (*P*_heterogeneity_ = 0.97, *I*^2^ = 0%).

The occurrence of esophagitis was included in five studies. In the POEM group, the incidence of esophagitis was 21.3% (29/136), whereas in the LHM group, it was 13.9% (16/115). There was no statistically significant difference observed (RR 1.43, *p* = 0.39; 95% CI: 0.63, 3.23) with low heterogeneity (*P*_heterogeneity_ = 0.16, *I*^2^ = 40%).

Regarding Los Angeles classification, the occurrence of grade A and B esophagitis was reported in five studies. The rate of esophagitis LA grades A and B was 17.7% (23/130) in the POEM group and 10.8% (12/111) in the LHM group. This difference was not statistically significant (RR 1.57, *p* = 0.29; 95% CI: 0.69, 3.58) with low heterogeneity (*P*_heterogeneity_ = 0.25, *I*^2^ = 26%).

The occurrence of grade C and D esophagitis was reported in four studies. The rate of esophagitis LA grades C and D was 6.5% (6/93) in the POEM group and 4.9% (4/82) in the LHM group. This difference was not statistically significant (RR 1.48, *p* = 0.51; 95% CI: 0.46, 4.70) with low heterogeneity (*P*_heterogeneity_ = 0.63, *I*^2^ = 0%).

All forest plots can be found in supplementary data.

## Discussion

In recent years, the treatment of achalasia has been the subject of several studies. While there are numerous therapeutic options available for this pathology, LHM and POEM are particularly noteworthy due to their sustained efficacy and a minimal occurrence of associated complications.

Regarding prior research, we identified seven meta-analyses that employed a methodology that was comparable to our own. The articles in question were compiled from 2015 to 2024, and they comprised studies that conducted direct comparisons between POEM and laparoscopic Heller myotomy fundoplication [31–37]. Nevertheless, the number of studies compared in these articles was comparatively lower than that of our meta-analysis, potentially impacting the outcomes that were obtained.

Regarding operative time, our research shows that it was significantly shorter in POEM when compared with LHM. This result defies the current body of literature, as the most recent meta-analyses on the subject determined that the operative time for both techniques was comparable [31–36]. According to some studies, the difference stems from the fact that POEM is an endoscopic procedure while LHM is a surgical intervention. Additionally, LHM requires a fundoplication, which lengthens the surgical procedure [12].

Our results show that there was no statistically significant difference in intraoperative complications between POEM and LHM. A significant proportion of these complications were injuries to the esophageal or gastric mucosa that occurred during the procedures and were promptly repaired. This is consistent with the available literature and demonstrates the safety of both treatments [31].

The clinical success, defined as an Eckardt score of three or lower, was significantly higher in the POEM group, when compared with the LHM group. The remaining meta-analyses did not report this outcome and chose to mention only the mean and standard deviation of the postoperative Eckardt score. Therefore, we consider this result highly relevant to the current literature.

In addition, some studies published the mean and standard deviation of the postoperative Eckardt score and we chose to report this outcome to complement the clinical success mentioned above. Therefore, there was no statistically significant difference between the POEM group and the LHM group. However, significant heterogeneity was identified in postoperative Eckardt score. Except for Zhang et al. and Park et al., who found that patients undergoing POEM had a lower postoperative Eckardt score than patients undergoing LHM, the majority of previous meta-analyses did not produce statistically significant differences regarding this outcome [34, 36].

Hence, our findings suggests that POEM could provide a more significant clinical improvement than LHM, especially during the initial months. Nevertheless, further research comparing the two procedures with longer follow-up periods is required to assess this outcome.

Regarding overall postoperative complications, they were comparable between both groups. These results were also observed in other meta-analyses and reveal that POEM is a safe procedure and comparable to LHM [31, 32]. Additionally, the CD classification has been applied in multiple studies to distinguish the severity of different postoperative complications. Most of the grade 3 complications documented in several studies were perforations or esophageal leakage requiring surgical intervention. Cases of sepsis, mediastinitis, and acute respiratory distress syndrome (all of which necessitated intensive care unit admission) constituted complications of grade 4 identified in three studies. There were no deaths related to the procedures.

Concerning reintervention due to clinical failure, it did not differ considerably between both procedures, although there was a trend to lower recurrence in the POEM group. The potential reduction in reinterventions due to recurrence observed with POEM may be attributed to the longer myotomy length that can be achieved with this technique [26].

Furthermore, our study found that postoperative pain was significantly lower in the POEM group. This finding is novel in the current literature, since the previous meta-analyses concluded that postoperative pain was comparable for both procedures [31, 32]. Several studies indicate that POEM is a less painful procedure than LHM because it doesn't require skin incisions [15, 24]. Nevertheless, this result was documented in two studies, as most of the studies incorporated in this meta-analysis assessed postoperative pain using distinct methodologies. To confirm this result, further research applying a standardized method of measurement is necessary.

In comparison to the LHM group, the POEM group exhibited a significantly reduced length of stay. Similar results have been reported by Martins et al. [33]. However, other meta-analyses did not show a significant difference regarding this outcome. Even though the costs associated with the procedures are not accounted for in this meta-analysis, this result is significant in this regard.

Nevertheless, numerous studies highlight that the main drawback of POEM is gastroesophageal reflux, since it doesn’t involve any antireflux procedure in contrast to surgical myotomy [1, 38, 39]. In this meta-analysis, postoperative gastroesophageal reflux was assessed using four outcomes. Following both procedures, postoperative GERD symptoms were comparable. Some studies applied the GERD HRQL questionnaire and found no statistically significant difference. Also, clinical records regarding esophagitis and the use of PPIs were similar between the two groups. So, our research did not identify any statistically significant differences between the two procedures regarding postoperative gastroesophageal reflux. With the exception of the findings of Marano et al., who reported a trend toward a significant reduction in the rate of symptomatic gastroesophageal reflux favoring LHM over POEM, this result is consistent with the other meta-analyses mentioned earlier [31].

While POEM lacks an associated anti-reflux mechanism, several studies suggest that the dissection executed during this technique is less disruptive and maintains a certain level of lower esophageal sphincter functionality, thereby decreasing the occurrence of gastroesophageal reflux [12, 25, 34, 40]. The previously mentioned endoscopic procedure involves the establishment of a submucosal tunnel via natural body orifice. There is no hiatal dissection during POEM compared to extensive dissection of the hiatus during a standard myotomy. This extensive dissection disrupts important ligaments of the esophagus, which are thought to contribute to the maintenance of the angle of His [6, 25, 40].

However, further research is needed to compare the two techniques directly and to determine the incidence of postoperative gastroesophageal reflux using the same method.

So, this meta-analysis encompasses the latest studies directly comparing LHM with POEM, presenting numerous results that could elucidate existing literature and guide future research. It is worth noting that twenty studies directly comparing both techniques were included, a significantly higher number compared to previous meta-analyses. Lastly, this study managed to demonstrate several differences between the procedures, such as superior clinical success in POEM, shorter operative time, shorter length of stay, and less postoperative pain in POEM.

There were some limitations to this study. The studies included were observational and non-randomized, the average follow-up time was less than a year, some outcomes analyzed showed high heterogeneity, and most studies included were from the United States, which may not represent global reality.

## Conclusion

In conclusion, this meta-analysis provides evidence that POEM, like LHM, is a safe and efficient treatment for achalasia. Nevertheless, POEM exhibits better results in terms of operative time, length of stay, postoperative pain, and a tendency towards lower recurrence. One noteworthy finding is that POEM exhibits superior clinical success compared to LHM during the initial months. Further multicenter randomized controlled trials featuring larger sample sizes are imperative to perform an all-encompassing comparison of the two techniques.

Hence, we believe that POEM may soon be regarded as the treatment of choice for achalasia.

### Supplementary Information

Below is the link to the electronic supplementary material.

Supplementary file1 (PDF 87 KB)

Supplementary file2 (PDF 3103 KB)
