# Supplementary material for: Achalasia: laparoscopic Heller myotomy with fundoplication versus peroral endoscopic myotomy—a systematic review and meta-analysis
Source: Esophagus. 2024 May 22;21(3):298–305. doi: 10.1007/s10388-024-01063-x (PMC11199208; doi:10.1007/s10388-024-01063-x)
Supplement: Supplementary file 1 — Supplementary file1 (PDF 87 KB) [file 10388_2024_1063_MOESM1_ESM.pdf]

Table 1 - Baseline characteristics of included patients:

	Age (LHM)		Age (POEM)		Sex (LHM)			Sex (POEM)		
**Author:**	**Mean**	**SD**	**Mean**	**SD**	**M**	**F**	**Pt**	**M**	**F**	**Pt**
**Hungness et al., 2012**	49.75	12.5	41.75	12.9	29	26	55	13	5	18
**Bhayani et al., 2014**	57	20	56	16	31	33	64	19	18	37
**Kumbhari et al., 2014**	51.6	17.9	58.3	18.8	13	13	26	29	20	49
**Chan et al., 2016**	38.4	14.3	48.2	15.17	11	12	23	12	21	33
**Docimo et al., 2016**	50.9	17.89	52.2	20.75	63	59	122	27	17	44
**Schneider et al., 2016**	53.25	6.87	58.75	1.78	12	13	25	13	12	25
**Ward et al., 2017**	61.5	19.3	63	17.9	14	10	24	25	16	41
**Leeds et al., 2017**	52.7	12.4	52	16.9	6	5	11	4	8	12
**de Pascale et al., 2017**	49.75	13.56	53.25	15.73	19	23	42	20	12	32
**Peng et al., 2017**	44.7	21.6	37.5	13	8	10	18	8	5	13
**Ramirez et al., 2017**	45	NA	50	NA	20	15	35	15	20	35
**Shea et al., 2019**	NA	NA	NA	NA	NA	NA	97	NA	NA	44
**Shemmeri et al., 2019**	NA	NA	NA	NA	NA	NA	114	NA	NA	71
**Wirsching et al., 2019**	57.25	17.89	58.5	10.37	12	16	28	11	12	23
**Ward et al., 2020**	54.1	16.8	56.9	16.7	13	33	46	19	35	54
**Attar et al., 2020**	58	19	64	16	19	14	33	62	64	126
**Kahaleh et al., 2020**	45.6	10.5	47.3	15.9	30	34	64	29	40	69
**Podboy et al., 2020**	57.93	2.4	59.18	2.4	10	33	43	22	33	55
**Trieu et al., 2021**	54.28	18.497	56	19.37	NA	NA	2850	NA	NA	580
**Shally et al., 2022**	59.5	5.6	57.25	4.09	NA	NA	25	NA	NA	33

F, female; LHM, Laparoscopic Heller Myotomy; M, male; NA, not available; POEM, Peroral Endoscopic Myotomy; Pt, patients; SD, standard deviation
