# Supplementary material for: Achalasia: laparoscopic Heller myotomy with fundoplication versus peroral endoscopic myotomy—a systematic review and meta-analysis
Source: Esophagus. 2024 May 22;21(3):298–305. doi: 10.1007/s10388-024-01063-x (PMC11199208; doi:10.1007/s10388-024-01063-x)
Supplement: Supplementary file 2 — Supplementary file2 (PDF 3103 KB) [file 10388_2024_1063_MOESM2_ESM.pdf]

Supplemental File – Forest Plots:

Study or Subgroup	POEM Mean	POEM SD	POEM Total	LHM Mean	LHM SD	LHM Total	Weight	Mean Difference IV, Random, 95% CI	Year
Hungness 2012	133.5	36.23	18	133.75	23.03	55	8.4%	−0.25 [−18.06, 17.56]	2012
Bhayani 2014	128.75	36.46	37	175	38.51	64	8.8%	−46.25 [−61.32, −31.18]	2014
Kumbhari 2014	148	67.54	49	262	35.84	26	7.4%	−114.00 [−137.40, −90.60]	2014
Chan 2016	97	40.8	33	127	30.1	23	8.3%	−30.00 [−48.58, −11.42]	2016
Schneider 2016	152.3	45.2	25	171.1	34.9	25	7.6%	−18.80 [−41.18, 3.58]	2016
Leeds 2017	136	64	12	154	37	11	4.6%	−18.00 [−60.30, 24.30]	2017
Pascale 2017	68.2	19.84	32	99.75	31.72	42	9.3%	−31.55 [−43.35, −19.75]	2017
Peng 2017	93.4	23.5	13	121.3	20.1	18	8.7%	−27.90 [−43.69, −12.11]	2017
Shea 2019	121	10.71	44	148.25	11.46	97	10.1%	−27.25 [−31.15, −23.35]	2019
Wirsching 2019	159.75	44.3	23	145.25	19.13	28	8.1%	14.50 [−4.94, 33.94]	2019
Attaar 2020	84	43	126	142	36	33	8.9%	−58.00 [−72.40, −43.60]	2020
Shally 2022	107.5	16.35	33	152.5	20.36	25	9.6%	−45.00 [−54.74, −35.26]	2022
**Total (95% CI)**			**445**			**447**	**100.0%**	**−33.80 [−46.07, −21.53]**	

Heterogeneity: $Tau^2 = 383.98$; $Chi^2 = 111.44$, df = 11 ($P < 0.00001$); $I^2 = 90\%$
Test for overall effect: Z = 5.40 ($P < 0.00001$)

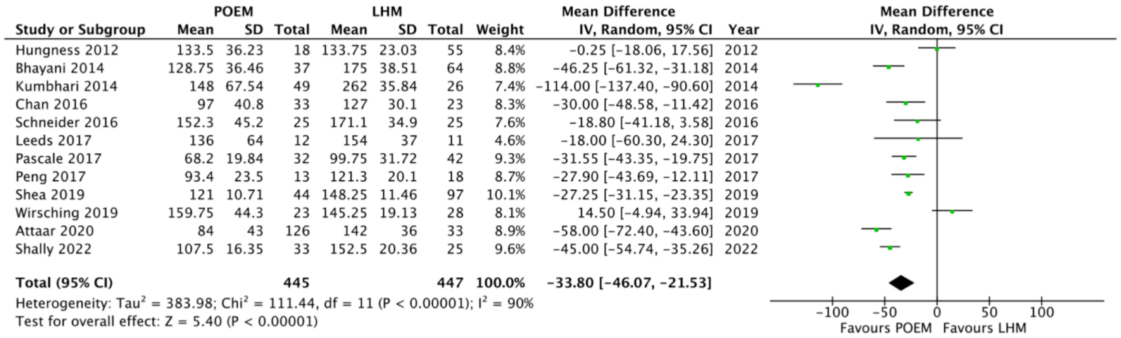

Figure 1 - Operative Time

Study or Subgroup	POEM Events	POEM Total	LHM Events	LHM Total	Weight	Risk Ratio M-H, Random, 95% CI	Year
Bhayani 2014	4	37	11	64	23.9%	0.63 [0.22, 1.83]	2014
Schneider 2016	7	25	3	25	18.0%	2.33 [0.68, 8.01]	2016
Pascale 2017	4	32	5	42	18.1%	1.05 [0.31, 3.60]	2017
Leeds 2017	2	12	3	11	10.8%	0.61 [0.12, 3.00]	2017
Ramirez 2017	2	35	2	35	7.6%	1.00 [0.15, 6.71]	2017
Wirsching 2019	2	23	1	28	5.0%	2.43 [0.24, 25.18]	2019
Attaar 2020	12	126	2	33	13.1%	1.57 [0.37, 6.68]	2020
Kahaleh 2020	7	69	0	64	3.4%	13.93 [0.81, 239.05]	2020
**Total (95% CI)**		**359**		**302**	**100.0%**	**1.21 [0.72, 2.04]**	
Total events	40		27				

Heterogeneity: $Tau^2 = 0.00$; $Chi^2 = 6.90$, df = 7 ($P = 0.44$); $I^2 = 0\%$
Test for overall effect: Z = 0.71 ($P = 0.48$)

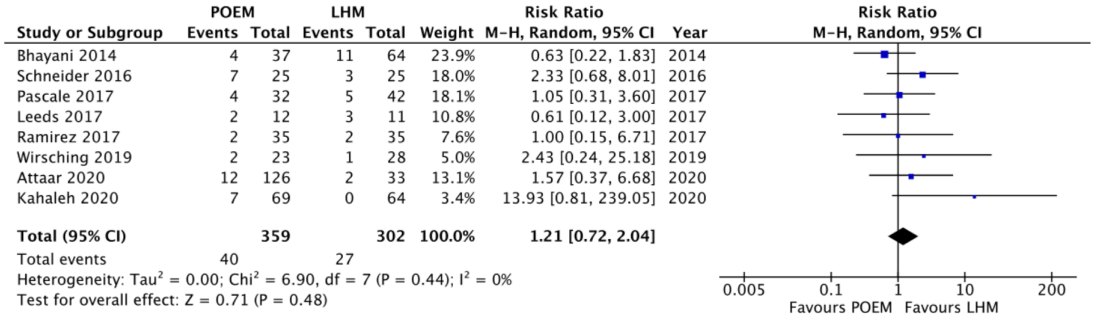

Figura 2 - Intraoperative Complications

Study or Subgroup	POEM Events	POEM Total	LHM Events	LHM Total	Weight	Risk Ratio M-H, Random, 95% CI	Year
Kumbhari 2014	48	49	21	26	8.5%	1.21 [1.00, 1.47]	2014
Schneider 2016	23	25	21	25	7.4%	1.10 [0.89, 1.35]	2016
Leeds 2017	9	11	6	9	1.1%	1.23 [0.72, 2.10]	2017
Peng 2017	9	12	12	15	1.8%	0.94 [0.62, 1.42]	2017
Ramirez 2017	31	35	27	35	6.7%	1.15 [0.93, 1.43]	2017
Ward 2017	40	41	20	23	11.4%	1.12 [0.95, 1.32]	2017
Shea 2019	22	30	34	52	3.7%	1.12 [0.84, 1.50]	2019
Shemmeri 2019	68	71	105	114	59.4%	1.04 [0.97, 1.12]	2019
**Total (95% CI)**		**274**		**299**	**100.0%**	**1.08 [1.02, 1.14]**	
Total events	250		246				

Heterogeneity: $Tau^2 = 0.00$; $Chi^2 = 4.18$, df = 7 ($P = 0.76$); $I^2 = 0\%$
Test for overall effect: Z = 2.59 ($P = 0.010$)

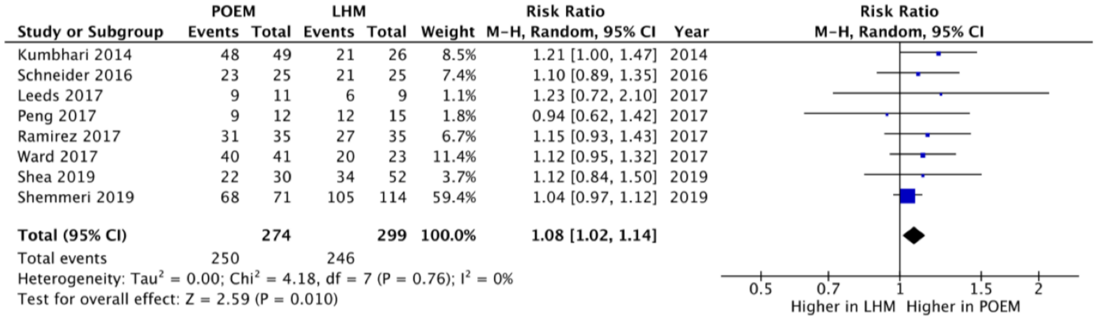

Figura 3 - Clinical Success

Study or Subgroup	POEM Mean	POEM SD	POEM Total	LHM Mean	LHM SD	LHM Total	Weight	Mean Difference IV, Random, 95% CI	Year
Schneider 2016	1.04	1.36	25	1	1.32	25	10.4%	0.04 [−0.70, 0.78]	2016
Peng 2017	2.6	1.5	12	2.8	1.3	15	8.6%	−0.20 [−1.27, 0.87]	2017
Ward 2017	0.9	1.6	41	1	1.3	24	10.5%	−0.10 [−0.81, 0.61]	2017
Leeds 2017	1.2	1.6	12	3	0.7	11	9.0%	−1.80 [−2.80, −0.80]	2017
Pascale 2017	3	2.42	32	1.25	0.69	42	9.7%	1.75 [0.89, 2.61]	2017
Wirsching 2019	0.5	0.53	9	0.5	0.52	22	12.0%	0.00 [−0.41, 0.41]	2019
Podboy 2020	0.8	1	55	0.7	1.1	43	11.9%	0.10 [−0.32, 0.52]	2020
Ward 2020	2.2	2.3	27	1.8	2.7	11	5.3%	0.40 [−1.42, 2.22]	2020
Attaar 2020	1	1.5	126	0.33	0.77	33	12.1%	0.67 [0.30, 1.04]	2020
Kahaleh 2020	1.84	1.9	69	3.6	2.4	64	10.4%	−1.76 [−2.50, −1.02]	2020
**Total (95% CI)**			**408**			**290**	**100.0%**	**−0.08 [−0.63, 0.46]**	

Heterogeneity: $Tau^2 = 0.60$; $Chi^2 = 62.34$, df = 9 ($P < 0.00001$); $I^2 = 86\%$
Test for overall effect: Z = 0.30 ($P = 0.76$)

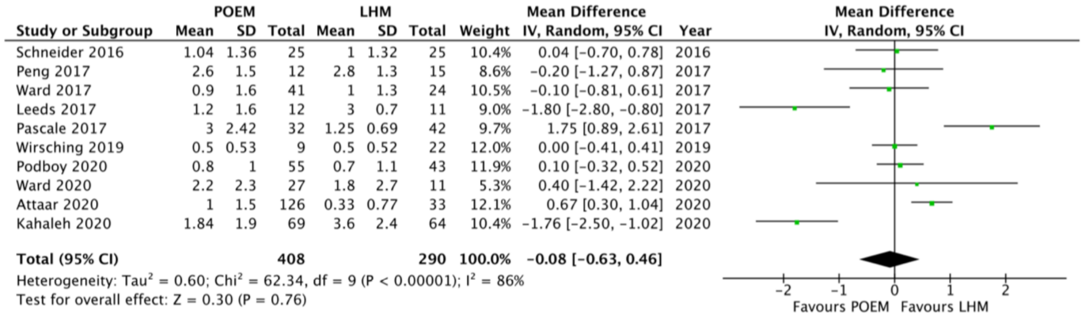

Figura 4 - Eckardt Score

Study or Subgroup	POEM Events	POEM Total	LHM Events	LHM Total	Weight	Risk Ratio M-H, Random, 95% CI	Year
Hungness 2012	4	18	8	55	9.5%	1.53 [0.52, 4.48]	2012
Bhayani 2014	1	37	1	64	2.4%	1.73 [0.11, 26.84]	2014
Kumbhari 2014	3	49	7	26	7.9%	0.23 [0.06, 0.81]	2014
Chan 2016	5	33	3	23	7.4%	1.16 [0.31, 4.39]	2016
Schneider 2016	1	25	1	25	2.4%	1.00 [0.07, 15.12]	2016
Leeds 2017	1	12	0	11	1.9%	2.77 [0.12, 61.65]	2017
Pascale 2017	0	32	2	42	2.0%	0.26 [0.01, 5.25]	2017
Peng 2017	5	13	1	18	4.0%	6.92 [0.91, 52.45]	2017
Ramirez 2017	1	35	1	35	2.4%	1.00 [0.07, 15.36]	2017
Shemmeri 2019	4	71	0	114	2.2%	14.38 [0.79, 263.05]	2019
Wirsching 2019	2	23	4	28	5.7%	0.61 [0.12, 3.03]	2019
Attaar 2020	9	126	6	33	10.7%	0.39 [0.15, 1.03]	2020
Kahaleh 2020	5	69	9	64	9.8%	0.52 [0.18, 1.46]	2020
Podboy 2020	10	55	12	43	13.3%	0.65 [0.31, 1.36]	2020
Trieu 2021	40	580	140	2850	18.4%	1.40 [1.00, 1.97]	2021
**Total (95% CI)**		**1178**		**3431**	**100.0%**	**0.89 [0.57, 1.40]**	
Total events	91		195				

Heterogeneity: $Tau^2 = 0.26$; $Chi^2 = 24.63$, df = 14 (P = 0.04); $I^2 = 43\%$
Test for overall effect: Z = 0.49 (P = 0.62)

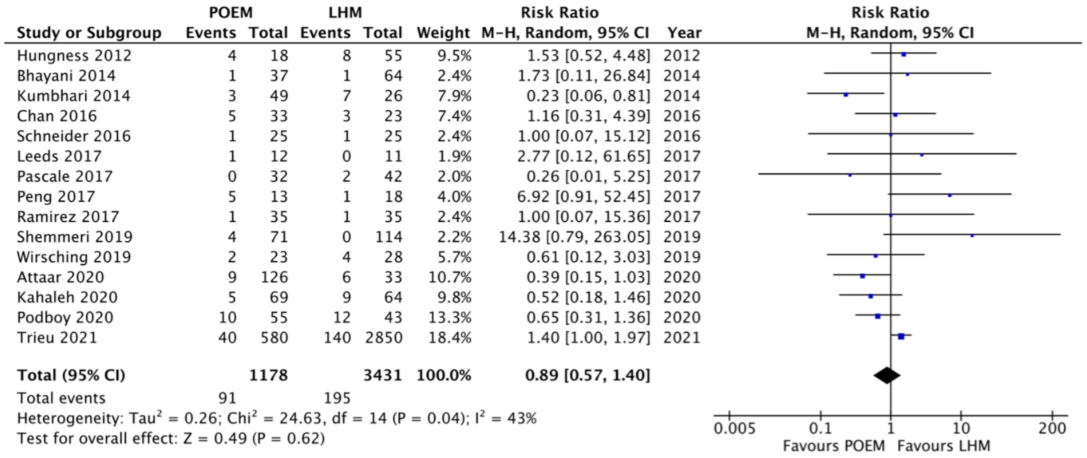

Figura 5 - Overall complications

Study or Subgroup	POEM Events	POEM Total	LHM Events	LHM Total	Weight	Risk Ratio M-H, Random, 95% CI	Year
Hungness 2012	3	17	7	54	10.6%	1.36 [0.39, 4.69]	2012
Kumbhari 2014	3	49	7	26	10.3%	0.23 [0.06, 0.81]	2014
Chan 2016	5	33	2	22	7.8%	1.67 [0.35, 7.84]	2016
Ramirez 2017	1	35	0	34	2.3%	2.92 [0.12, 69.20]	2017
Peng 2017	4	12	1	18	4.9%	6.00 [0.76, 47.36]	2017
Wirsching 2019	2	23	4	28	7.4%	0.61 [0.12, 3.03]	2019
Podboy 2020	8	55	10	41	16.2%	0.60 [0.26, 1.38]	2020
Attaar 2020	3	120	3	30	7.8%	0.25 [0.05, 1.18]	2020
Kahaleh 2020	3	67	6	61	9.5%	0.46 [0.12, 1.74]	2020
Trieu 2021	20	560	90	2800	23.3%	1.11 [0.69, 1.79]	2021
**Total (95% CI)**		**971**		**3114**	**100.0%**	**0.78 [0.47, 1.30]**	
Total events	52		130				

Heterogeneity: $Tau^2 = 0.22$; $Chi^2 = 14.79$, df = 9 (P = 0.10); $I^2 = 39\%$
Test for overall effect: Z = 0.95 (P = 0.34)

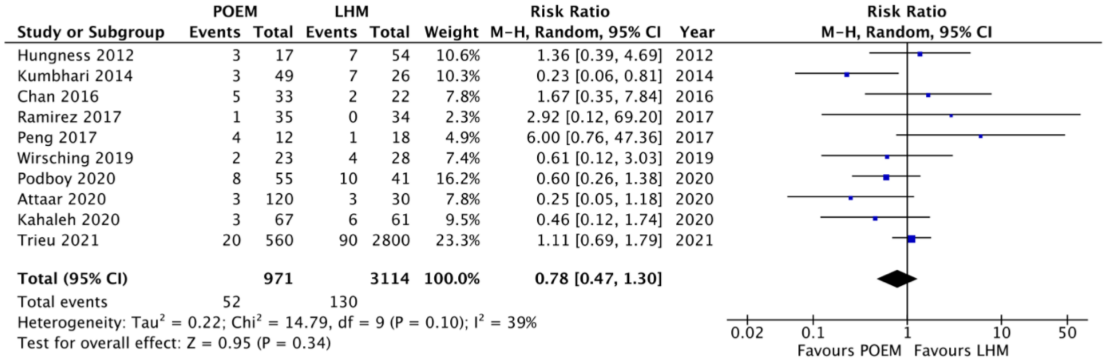

Figura 6 - Grades I and II in the CD classification

Study or Subgroup	POEM Events	POEM Total	LHM Events	LHM Total	Weight	Risk Ratio M-H, Random, 95% CI	Year
Hungness 2012	1	15	1	48	5.2%	3.20 [0.21, 48.12]	2012
Bhayani 2014	1	37	1	64	5.1%	1.73 [0.11, 26.84]	2014
Schneider 2016	1	25	1	25	5.2%	1.00 [0.07, 15.12]	2016
Chan 2016	0	28	1	21	3.9%	0.25 [0.01, 5.91]	2016
Peng 2017	1	9	0	17	4.0%	5.40 [0.24, 120.57]	2017
Ramirez 2017	0	34	1	35	3.8%	0.34 [0.01, 8.13]	2017
Pascale 2017	0	32	1	41	3.8%	0.42 [0.02, 10.08]	2017
Leeds 2017	1	12	0	11	4.0%	2.77 [0.12, 61.65]	2017
Shemmeri 2019	4	71	0	114	4.5%	14.38 [0.79, 263.05]	2019
Kahaleh 2020	2	66	3	58	12.5%	0.59 [0.10, 3.39]	2020
Podboy 2020	0	47	1	32	3.8%	0.23 [0.01, 5.45]	2020
Attaar 2020	0	117	1	28	3.8%	0.08 [0.00, 1.96]	2020
Trieu 2021	5	545	20	2730	40.3%	1.25 [0.47, 3.32]	2021
**Total (95% CI)**		**1038**		**3224**	**100.0%**	**1.07 [0.57, 1.98]**	
Total events	16		31				

Heterogeneity: $Tau^2 = 0.00$; $Chi^2 = 10.87$, df = 12 (P = 0.54); $I^2 = 0\%$
Test for overall effect: Z = 0.20 (P = 0.84)

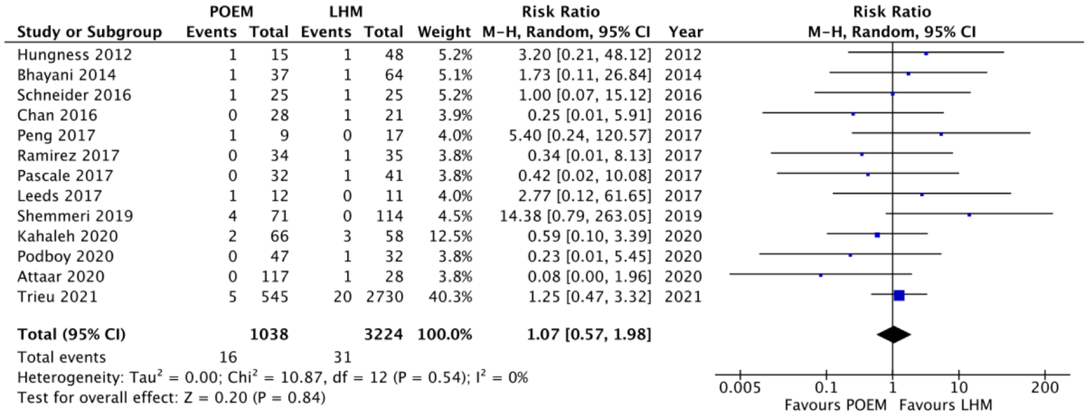

Figura 7 – Grade III in the CD classification

Study or Subgroup	POEM Events	POEM Total	LHM Events	LHM Total	Weight	Risk Ratio M-H, Random, 95% CI	Year
Pascale 2017	0	32	1	41	17.5%	0.42 [0.02, 10.08]	2017
Podboy 2020	0	47	1	32	17.5%	0.23 [0.01, 5.45]	2020
Trieu 2021	15	555	30	2740	65.0%	2.47 [1.34, 4.56]	2021
**Total (95% CI)**		**634**		**2813**	**100.0%**	**1.20 [0.26, 5.48]**	
Total events	15		32				

Heterogeneity: $Tau^2 = 0.83$; $Chi^2 = 3.20$, df = 2 (P = 0.20); $I^2 = 37\%$
Test for overall effect: Z = 0.23 (P = 0.82)

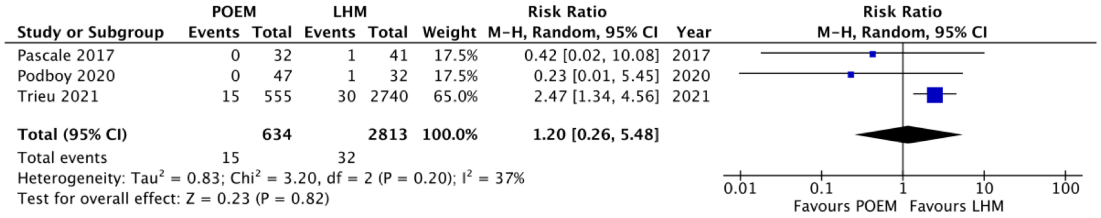

Figura 8 - Grade IV in the CD classification

Study or Subgroup	POEM Events	POEM Total	LHM Events	LHM Total	Weight	Risk Ratio M-H, Random, 95% CI	Year
Kumbhari 2014	0	49	2	26	2.7%	0.11 [0.01, 2.17]	2014
Chan 2016	0	33	1	23	2.5%	0.24 [0.01, 5.53]	2016
Ramirez 2017	10	35	8	35	23.1%	1.25 [0.56, 2.79]	2017
Shemmeri 2019	3	71	3	114	8.8%	1.61 [0.33, 7.74]	2019
Wirsching 2019	1	23	1	28	3.3%	1.22 [0.08, 18.41]	2019
Kahaleh 2020	10	69	23	64	28.4%	0.40 [0.21, 0.78]	2020
Podboy 2020	12	55	20	44	31.2%	0.48 [0.26, 0.87]	2020
**Total (95% CI)**		**335**		**334**	**100.0%**	**0.62 [0.37, 1.02]**	
Total events	36		58				

Heterogeneity: $Tau^2 = 0.12$; $Chi^2 = 8.49$, df = 6 (P = 0.20); $I^2 = 29\%$
Test for overall effect: Z = 1.87 (P = 0.06)

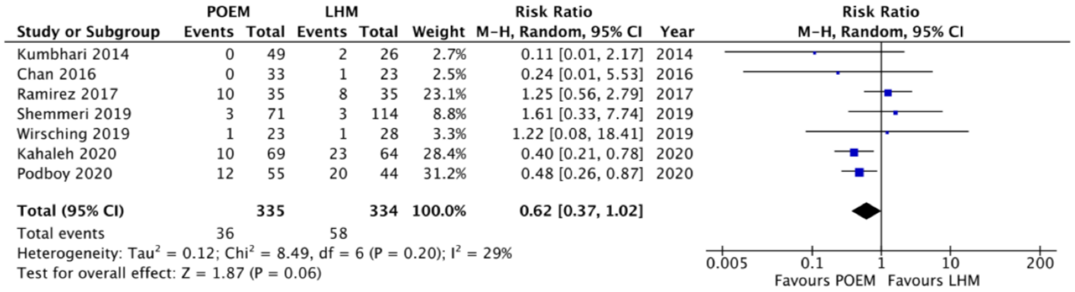

Figura 9 - Reintervention rate

Study or Subgroup	POEM Mean	POEM SD	POEM Total	LHM Mean	LHM SD	LHM Total	Weight	Mean Difference IV, Random, 95% CI	Year
Ward 2017	2.6	5.5	41	3.1	2.1	24	18.1%	−0.50 [−2.38, 1.38]	2017
Attaar 2020	1.33	2.25	126	2.33	2.32	33	81.9%	−1.00 [−1.88, −0.12]	2020
**Total (95% CI)**			**167**			**57**	**100.0%**	**−0.91 [−1.71, −0.11]**	

Heterogeneity: $Tau^2 = 0.00$; $Chi^2 = 0.22$, df = 1 (P = 0.64); $I^2 = 0\%$
Test for overall effect: Z = 2.23 (P = 0.03)

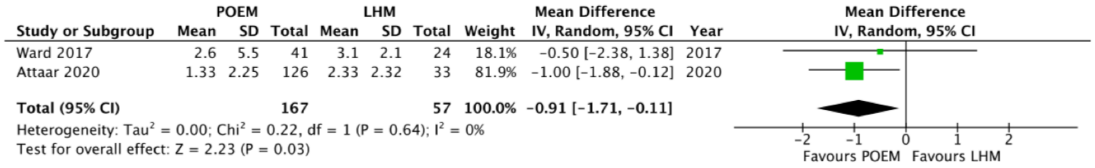

Figura 10 - Postoperative pain

Study or Subgroup	POEM Mean	POEM SD	POEM Total	LHM Mean	LHM SD	LHM Total	Weight	Mean Difference IV, Random, 95% CI	Year
Hungness 2012	4	3.29	18	5.5	3.95	55	1.9%	−1.50 [−3.34, 0.34]	2012
Bhayani 2014	1.1	0.6	37	2.5	1.9	64	8.3%	−1.40 [−1.90, −0.90]	2014
Kumbhari 2014	3.3	1.9	49	3.2	2.3	26	4.5%	0.10 [−0.93, 1.13]	2014
Chan 2016	3.15	1.1	33	3.4	1.4	23	6.8%	−0.25 [−0.93, 0.43]	2016
Docimo 2016	1.3	0.43	44	2.32	1.11	122	10.6%	−1.02 [−1.25, −0.79]	2016
Leeds 2017	1.6	1.2	12	2	1.9	11	3.2%	−0.40 [−1.71, 0.91]	2017
Pascale 2017	4.25	1.69	32	3.25	1.15	42	6.8%	1.00 [0.32, 1.68]	2017
Peng 2017	4	0.3	13	5	0.55	18	10.1%	−1.00 [−1.30, −0.70]	2017
Ward 2017	2	3.3	41	2.5	2.6	24	2.8%	−0.50 [−1.95, 0.95]	2017
Wirsching 2019	1.25	0.25	23	1.75	0.75	28	10.1%	−0.50 [−0.80, −0.20]	2019
Attaar 2020	0.67	0.75	126	1.67	0.77	33	10.1%	−1.00 [−1.29, −0.71]	2020
Podboy 2020	1.7	1.7	55	2.6	2.5	43	5.4%	−0.90 [−1.77, −0.03]	2020
Trieu 2021	3.64	6	580	3.08	4.7	2850	8.2%	0.56 [0.04, 1.08]	2021
Shally 2022	0.72	0.25	33	1.33	0.23	25	11.2%	−0.61 [−0.73, −0.49]	2022
**Total (95% CI)**			**1096**			**3364**	**100.0%**	**−0.55 [−0.83, −0.27]**	

Heterogeneity: $Tau^2 = 0.18$; $Chi^2 = 77.79$, df = 13 (P < 0.00001); $I^2 = 83\%$
Test for overall effect: Z = 3.85 (P = 0.0001)

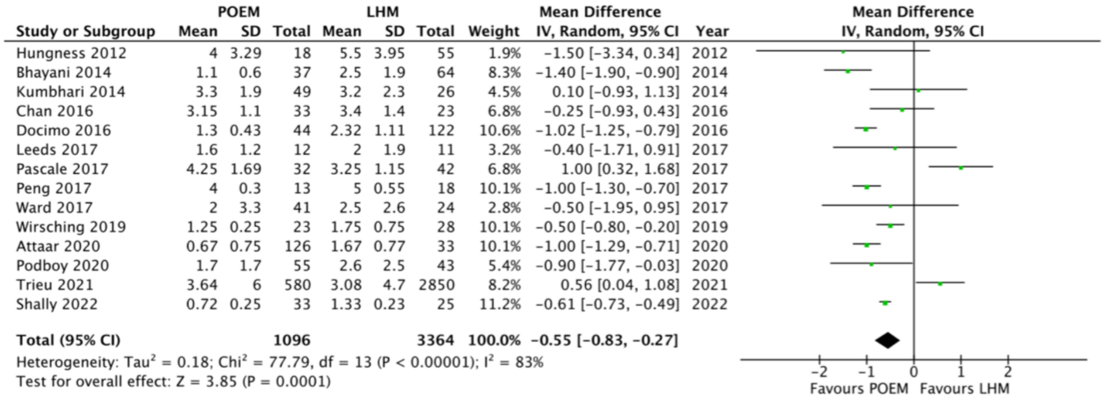

Figura 11 - Length of stay

Study or Subgroup	POEM Events	POEM Total	LHM Events	LHM Total	Weight	Risk Ratio M-H, Random, 95% CI	Year
Bhayani 2014	4	37	3	63	6.1%	2.27 [0.54, 9.59]	2014
Chan 2016	5	33	6	23	11.2%	0.58 [0.20, 1.68]	2016
Pascale 2017	4	32	4	42	7.4%	1.31 [0.36, 4.85]	2017
Peng 2017	1	12	1	15	1.8%	1.25 [0.09, 17.98]	2017
Ramirez 2017	7	35	6	35	13.0%	1.17 [0.44, 3.12]	2017
Podboy 2020	22	55	20	43	60.6%	0.86 [0.55, 1.36]	2020
**Total (95% CI)**		**204**		**221**	**100.0%**	**0.94 [0.66, 1.34]**	
Total events	43		40				

Heterogeneity: $Tau^2 = 0.00$; $Chi^2 = 2.89$, df = 5 (P = 0.72); $I^2 = 0\%$
Test for overall effect: Z = 0.32 (P = 0.75)

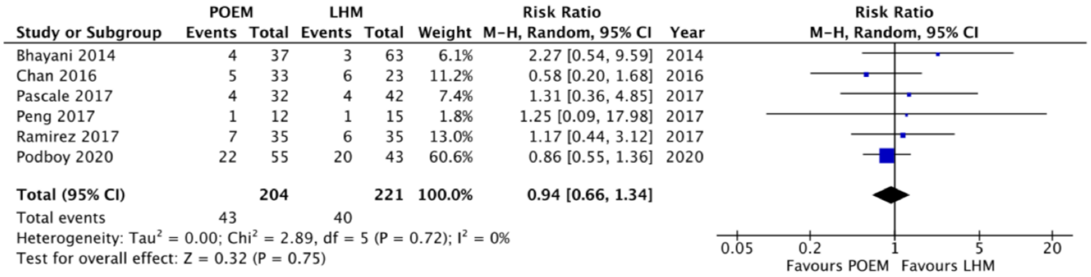

Figura 12 - Occurrence of GERD symptoms

Study or Subgroup	POEM Mean	POEM SD	POEM Total	LHM Mean	LHM SD	LHM Total	Weight	Mean Difference IV, Random, 95% CI	Year
Schneider 2016	5.35	7.39	25	4.15	5.47	25	26.9%	1.20 [−2.40, 4.80]	2016
Shea 2019	5.96	7.9	30	6.3	7.4	52	29.0%	−0.34 [−3.81, 3.13]	2019
Ward 2020	14.1	14.9	27	10.6	9	11	5.8%	3.50 [−4.24, 11.24]	2020
Attaar 2020	6.4	6.5	78	3.7	4.7	12	38.2%	2.70 [−0.33, 5.73]	2020
**Total (95% CI)**			**160**			**100**	**100.0%**	**1.46 [−0.41, 3.33]**	

Heterogeneity: $Tau^2 = 0.00$; $Chi^2 = 1.97$, df = 3 (P = 0.58); $I^2 = 0\%$
Test for overall effect: Z = 1.53 (P = 0.13)

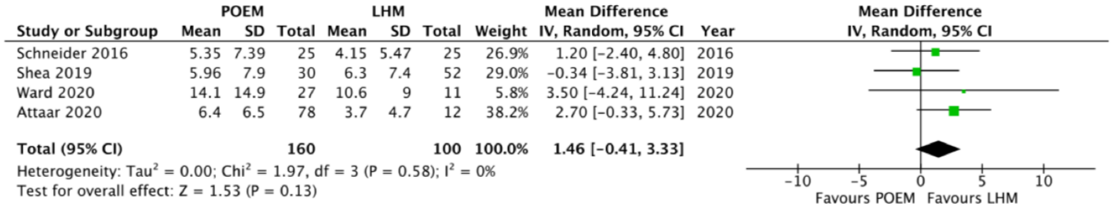

Figura 13 - GERD HRQL

Study or Subgroup	POEM Events	POEM Total	LHM Events	LHM Total	Weight	Risk Ratio M-H, Random, 95% CI	Year
Kumbhari 2014	19	49	12	26	43.0%	0.84 [0.49, 1.45]	2014
Chan 2016	3	33	3	23	5.6%	0.70 [0.15, 3.15]	2016
Peng 2017	1	12	1	15	1.8%	1.25 [0.09, 17.98]	2017
Ramirez 2017	8	35	7	35	15.7%	1.14 [0.46, 2.81]	2017
Podboy 2020	16	55	13	43	33.8%	0.96 [0.52, 1.78]	2020
**Total (95% CI)**		**184**		**142**	**100.0%**	**0.92 [0.64, 1.31]**	
Total events	47		36				

Heterogeneity: Tau² = 0.00; Chi² = 0.54, df = 4 (P = 0.97); I² = 0%
Test for overall effect: Z = 0.46 (P = 0.65)

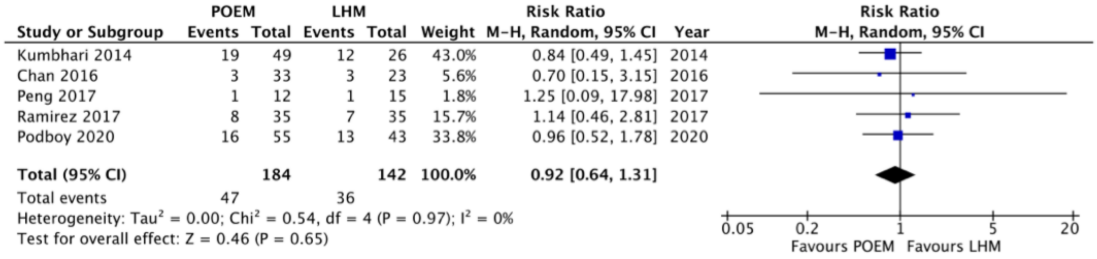

Figura 14 - Use of proton pump inhibitors

Study or Subgroup	POEM Events	POEM Total	LHM Events	LHM Total	Weight	Risk Ratio M-H, Random, 95% CI	Year
Schneider 2016	7	13	6	19	34.2%	1.71 [0.74, 3.92]	2016
Pascale 2017	8	20	1	20	12.8%	8.00 [1.10, 58.19]	2017
Ramirez 2017	1	21	1	22	7.7%	1.05 [0.07, 15.69]	2017
Podboy 2020	2	55	5	43	17.5%	0.31 [0.06, 1.53]	2020
Ward 2020	11	27	3	11	27.8%	1.49 [0.51, 4.34]	2020
**Total (95% CI)**		**136**		**115**	**100.0%**	**1.43 [0.63, 3.23]**	
Total events	29		16				

Heterogeneity: Tau² = 0.32; Chi² = 6.64, df = 4 (P = 0.16); I² = 40%
Test for overall effect: Z = 0.87 (P = 0.39)

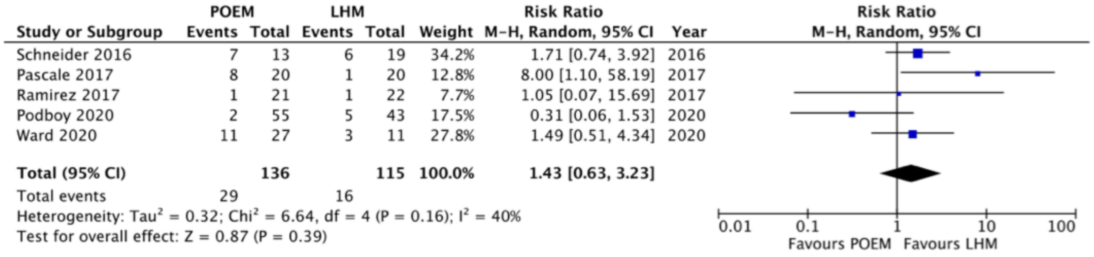

Figura 15 – Esophagitis

Study or Subgroup	POEM Events	POEM Total	LHM Events	LHM Total	Weight	Risk Ratio M-H, Random, 95% CI	Year
Schneider 2016	5	11	4	17	33.7%	1.93 [0.66, 5.65]	2016
Ramirez 2017	1	21	1	22	8.3%	1.05 [0.07, 15.69]	2017
Pascale 2017	7	19	1	20	14.0%	7.37 [1.00, 54.39]	2017
Podboy 2020	2	55	4	42	19.0%	0.38 [0.07, 1.99]	2020
Ward 2020	8	24	2	10	25.0%	1.67 [0.43, 6.51]	2020
**Total (95% CI)**		**130**		**111**	**100.0%**	**1.57 [0.69, 3.58]**	
Total events	23		12				

Heterogeneity: Tau² = 0.23; Chi² = 5.38, df = 4 (P = 0.25); I² = 26%
Test for overall effect: Z = 1.07 (P = 0.29)

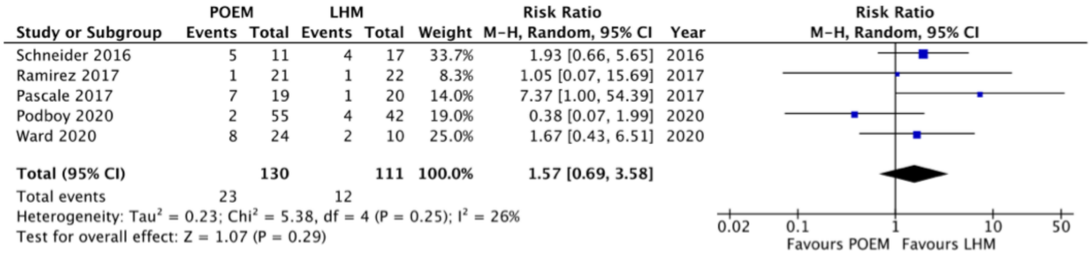

Figura 16 - Esophagitis LA grades A and B

Study or Subgroup	POEM Events	POEM Total	LHM Events	LHM Total	Weight	Risk Ratio M-H, Random, 95% CI	Year
Schneider 2016	2	8	2	15	43.2%	1.88 [0.32, 10.92]	2016
Pascale 2017	1	13	0	19	13.7%	4.29 [0.19, 97.73]	2017
Podboy 2020	0	53	1	39	13.3%	0.25 [0.01, 5.90]	2020
Ward 2020	3	19	1	9	29.8%	1.42 [0.17, 11.83]	2020
**Total (95% CI)**		**93**		**82**	**100.0%**	**1.48 [0.46, 4.70]**	
Total events	6		4				

Heterogeneity: Tau² = 0.00; Chi² = 1.75, df = 3 (P = 0.63); I² = 0%
Test for overall effect: Z = 0.66 (P = 0.51)

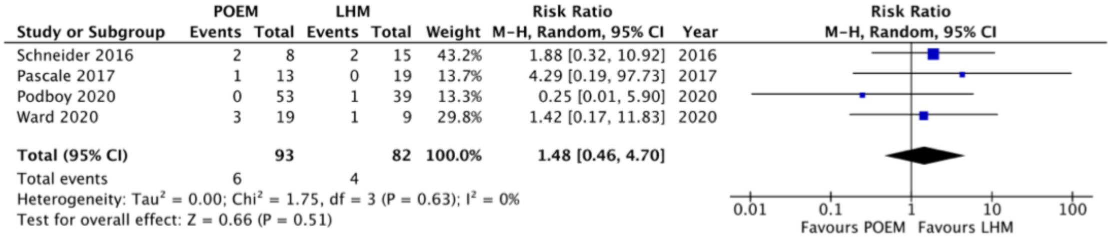

Figura 17 - Esophagitis LA grades C and D
